# Analysis of the operational status of the three-level referral system for urologic ultrasound screening and risk factors for renal pelvic dilatation in high-risk children

**DOI:** 10.3389/fped.2023.1162952

**Published:** 2023-04-24

**Authors:** Baowei Ji, Yinv Gong, Ying Zhang, Yun Li, Yihui Zhai, Yinghua Sun, Xiang Wang, Lishan Jia, Hong Xu, Qian Shen

**Affiliations:** ^1^ Department of Nephrology, Children’s Hospital of Fudan University, Shanghai, China; ^2^ Department of Rheumatology, Children’s Hospital of Fudan University, Shanghai, China; ^3^ Department of Child Health, Minhang Maternal and Child Health Hospital, Shanghai, China; ^4^ Department of Ultrasonography, Children’s Hospital of Fudan University, Shanghai, China; ^5^ Department of Urology, Children’s Hospital of Fudan University, Shanghai, China; ^6^ Department of Pediatrics, Taicang Affiliated Hospital of Soochow University, The First People's Hospital of Taicang, Suzhou, China

**Keywords:** ultrasound screening, renal pelvis dilatation, risk factors, high-risk children, patient transfer

## Abstract

**Background:**

Congenital Anomalies of the Kidney and Urinary Tract (CAKUT) are the primary cause of end-stage renal disease in children, early diagnosis and treatment can significantly improve the kidney function. Among CAKUT, renal pelvis dilatation (RPD) due to various causes has the highest detection rate, which can be detected early by postnatal ultrasound screening. Since 2010, the Children's Hospital of Fudan University (CHFU), together with the Minhang District Maternal and Child Health Hospital (MCH) and Community Health Centres (CHCs) of Minhang District has created a three-level referral system for urological ultrasound screening. This study aims to describe the operation of a three-level referral system for ultrasound screening of CAKUT and to select risk factors of RPD in high-risk children.

**Methods:**

The operation of the three-level referral system was assessed by analyzing the screening volume, screening rate, referral rate, and follow-up rate; risk factors of RPD in high-risk children were selected by chi-square test and multivariate logistic regression.

**Results:**

A total of 16,468 high-risk children were screened in ten years, and the screening volume was maintained at about 1,500 cases per year; the screening rate showed a linear increase, from 36.8% in 2010 to 98.2% in 2019; the referral rate from the CHCs to the MCH was 89.9% significantly higher after 2015 than that of 84.7% from 2010 to 2015; the follow-up rate after 2015 was 71.0% significantly higher than that of 46.3% from 2010 to 2015. Multivariate logistic regression analysis showed that the risk of RPD was 1.966 times higher in males than in females, and the risk of moderate to severe RPD was 2.570 times higher in males than in females; the risk of RPD in preterm children was 1.228 times higher than that of full-term children; and the risk of RPD was 1.218 times higher in twins than in singles.

**Conclusions:**

The screening volume of the three-level referral system has remained stable over a decade, with significantly higher screening, referral, and follow-up rates. Males, preterm, and twins are risk factors of RPD in high-risk children; males are also risk factors for moderate to severe RPD in high-risk children.

## 1. Introduction

CAKUT are a series of anatomical abnormalities of the kidney, ureter and urethra and are common birth defects with an incidence of 3–6/1,000 (1–3). Moreover, CAKUT is the leading cause of end-stage renal disease in children and adolescents and is also one of the leading causes of neonatal mortality (4, 5). Early postnatal ultrasound screening could detect the vast majority of CAKUT and is an important screening and follow-up tool (6, 7). Early intervention to detect abnormalities can have a significant improvement in renal function (8, 9), which has been carried out in Japan, Germany, Italy and the Czech Republic. Among CAKUT, RPD of various causes has the highest detection rate, and previous studies have shown that mild RPD is mostly spontaneous, however, moderate to severe RPD has a higher probability of diagnosing severe obstructive nephropathy and requiring surgical intervention (10, 11). In 2010, the National Children's Medical Center, CHFU, in collaboration with the MCH of Minhang District and CHCs of Minhang District, created a three-level referral system for urinary postnatal ultrasound screening of CAKUT in Minhang District, Shanghai (11), and it is necessary to note that routine prenatal screening was not performed in this study. In this study, we evaluate the operational efficiency of this system during the past ten years by analyzing the screening volume, screening rate, referral rate and follow-up rate; and screen risk factors for the occurrence of RPD in high-risk children by analyzing clinical factors during maternity and neonatal periods, which will help to promote a three-level referral system for postnatal urological screening of high-risk children and to identify risk factors for RPD in high-risk children.

## 2. Article types

Original Research articles.

## 3. The subjects and statistics

### 3.1. Subjects

**Figure 1:**
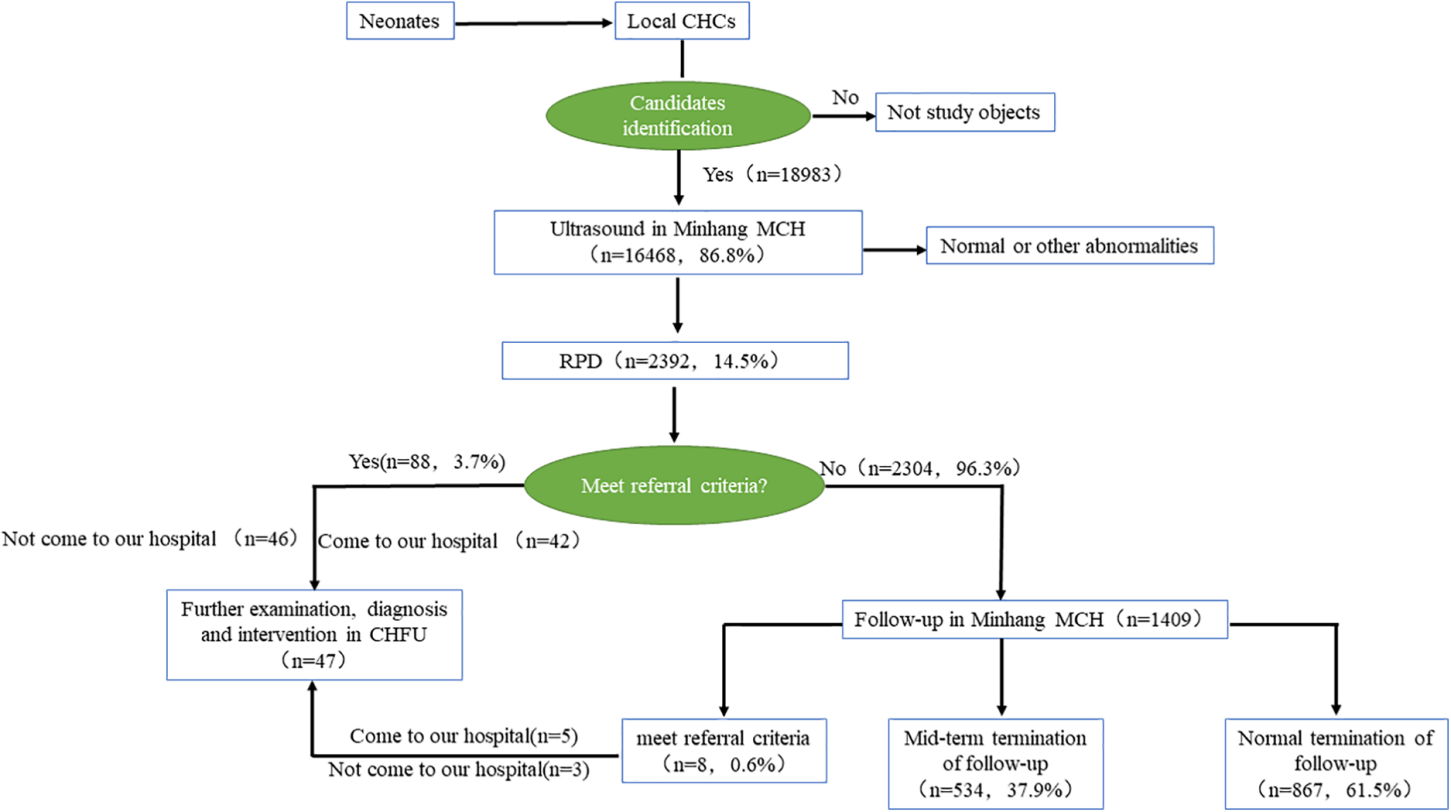
Flowchart and general information of research objects. CHCs, community health centers; MCH, maternal and child health hospital; RPD, renal pelvis dilatation; CHFU, children's hospital of fudan university.

The study was approved by the Research Ethical Committee of Children's Hospital of Fudan University 2012-174. This study was conducted to evaluate the operation of the three-level referral system for urological ultrasound screening of high-risk children in Minhang District, Shanghai over the past ten years. The three-level referral system was used to identify high-risk children in the Minhang district CHCs and transfer them to the Minhang MCH for a series of examinations, and those who met the referral criteria were referred to the CHFU for further examination and follow-up, while those who did not meet the referral criteria continued to be followed up at the Minhang MCH (Figure 1). Detailed information on the subjects of ultrasound screening, the definition of high-risk children and the criteria for determining abnormalities in ultrasound screening can be found in the literature (11). In brief, children at high risk of ongoing health issues following birth were defined as Low birth weight, Macrosomia, Preterm, Twins, Mother's age at childbirth >35 years, Gestational diabetes, Hypertension during pregnancy, History of asphyxia resuscitation, History of infection or drug application within the mother's third trimester of pregnancy, Post-term infants, Intracranial hemorrhage or intracranial infection, Congenital diseases (including birth defects), Dystocia, Hyperbilirubinemia, and they were referred through the three-level referral system for evaluation, treatment, and follow-up as necessary. A proportion of these children underwent postnatal ultrasound screening for abnormalities including a search for urological abnormalities. It is the subgroup of children from the “at risk” population that underwent ultrasound urologic screening that are the subjects of this study.

### 3.2. Statistics

The data analysis was performed using SPSS 20.0 statistical software. Count data were expressed as *n* (%), and comparisons between two groups were made by *χ*^2^ test or Fisher's exact probability method, and factors of statistical significance were further analyzed by multivariate logistic regression. A linear trend between year and screening rate was examined using the *χ*^2^ test. *P* < 0.05 was considered a statistically significant difference.

## 4. Result

### 4.1. Operation status of the three-level referral system for urological ultrasound screening

**Figure 2:**
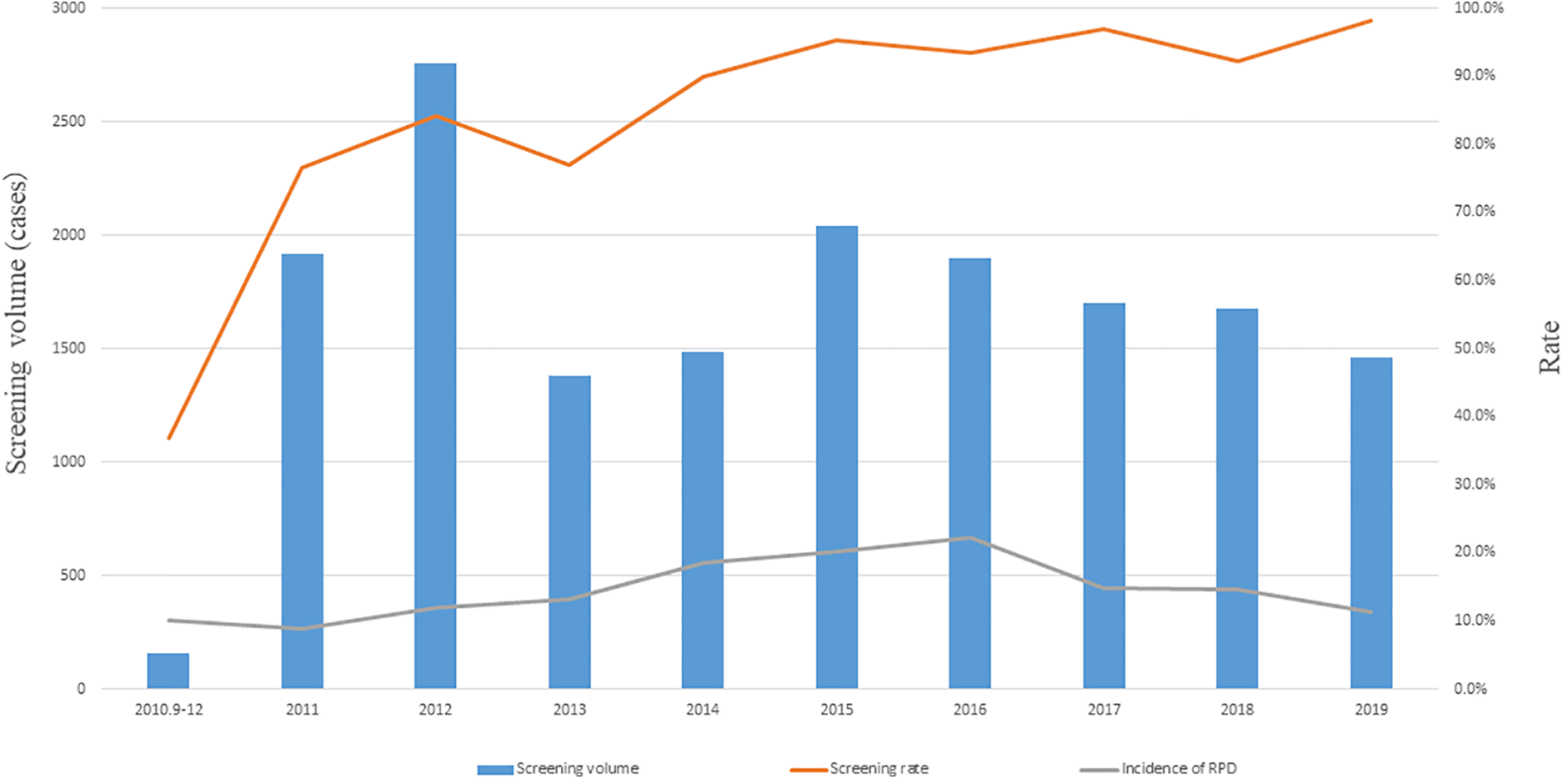
Screening volume, screening rate and incidence of RPD, from September 2010 to December 2019.

The volume of urological ultrasound screening was highest in 2012, at 2,757 cases, and the others remained around 1,500 cases per year; the urological ultrasound screening rate showed a linear trend year by year, showing a linear increasing trend (*Z* = 488.614, *P* < 0.001), and the screening rate has remained above 90% after 2014, reaching 98.2% in 2019; the incidence of RPD fluctuated to some extent, with the highest in 2016, at 22.3%, and remaining maintained at about 15% per year (Figure 2).

From 2015 to 2017, after taking the opportunity of the *three-year action plan for strengthening the public health system in Shanghai*, the CHFU and the Shanghai MCH integrated medical resources, provided relevant training and education to primary care physicians and parents of children, the referral rate and the follow-up rate after 2015 were significantly higher than those from 2010 to 2015, and the three-level referral system for urological ultrasound screening was consolidated and improved (Table 1).

**Table 1:** Comparison of referral rates and follow-up rates between different time periods.

	CHCs referrals	MCH screened	Referral rates	positive screenings	follow-up	Follow-up rates
2010–2015	11.500	9.740	84.7%	1.210	560	46.3%
2016–2019	7.483	6.728	89.9%	1.253	890	71.0%
*P*-value	–	–	<0.001	–	–	<0.001

### 4.2. General information

Between September 2010 and December 2019, a total of 18.983 high-risk children were referred to the MCH by CHCs in Minhang District, Shanghai, and 16.468 children were actually screened with urinary ultrasound, with an overall screening rate of 86.8% (16.468/18.983), 9.021 males and 7.447 females, male: female = 1.2:1, and other general information is shown in Table 2.

**Table 2:** General information on high-risk children undergoing ultrasound screening.

	Cases (%)
Gender	
Male	9.021 (54.8%)
Female	7.447 (45.2%)
Age at screening (months)	
0–1	2.023 (12.3%)
1–2	10.343 (62.8%)
2–3	2.224 (13.5%)
>3	1.878 (11.4%)
Birth weight (g)	
<2,500	7.229 (43.9%)
2,500–4,000	7.034 (42.7%)
>4,000	2.205 (13.4%)
Factors associated with high-risk children	
Preterm	10.289 (62.5%)
Twins	3.568 (21.7%)
Mother's age at childbirth >35 years	1.109 (6.7%)
Gestational diabetes	7 (0.04%)
Hypertension during pregnancy	21 (0.1%)
History of asphyxia resuscitation	253 (1.5%)
History of infection or drug application within the mother's third trimester of pregnancy	5 (0.03%)
Post-term infants	19 (0.1%)
Intracranial hemorrhage or intracranial infection	21 (0.1%)
Congenital diseases (including birth defects)	10 (0.06%)
Dystocia	86 (0.5%)
Hyperbilirubinemia	60 (0.4%)

### 4.3. Analysis of risk factors for renal pelvic dilatation in high-risk children

The results of comparison between groups showed that differences were statistically significant for males, preterm, low birth weight, macrosomia, twins, and dystocia (Table 3).

**Table 3:** Comparison of risk factors between normal and RPD groups, and moderate to severe RPD groups.

Projects	Normal group (*n* = 14.005)	RPD group (*n* = 2.392)	Moderate to severe RPD group (*n* = 267)	*P*1-value^1^	*P*2-value^2^
Gender					
Male	7.372 (52.6%)	1.623 (67.9%)	203 (76.0%)	<0.001	<0.001
Female	6.633 (47.4%)	769 (32.1%)	64 (24.0%)		
Gestational Week of Birth					
Preterm birth	8.577 (61.2%)	1.662 (69.5%)	154 (57.7%)	<0.001	0.254
Full-term	5.428 (38.8%)	730 (30.5%)	113 (42.3%)		
Low birth weight	6.074 (43.4%)	1.105 (46.2%)	88 (33.0%)	0.010	0.001
Macrosomia	1.988 (14.2%)	212 (8.9%)	47 (17.6%)	<0.001	0.129
Maternal age at childbirth					
19–34	13.040 (93.1%)	2.251 (94.1%)	250 (93.6%)	0.079	0.890
≥35	965 (6.9%)	141 (5.9%)	17 (6.4%)		
Single/Twins					
Single	11.061 (79.0%)	1.788 (74.7%)	200 (74.9%)	<0.001	0.110
Twins	2.944 (21.0%)	604 (25.3%)	67 (25.1%)		
History of asphyxia resuscitation	224 (1.6%)	29 (1.2%)	3 (1.1%)	0.181	0.803
Dystocia	79 (0.6%)	5 (0.2%)	1 (0.4%)	0.020	1.000
Intracranial hemorrhage or intracranial infection	18 (0.1%)	3 (0.1%)	0	1.000	–
Hyperbilirubinemia	55 (0.4%)	5 (0.2%)	0	0.201	–
Post-term infants	18 (0.1%)	1 (0.04%)	0	0.344	–
Hypertension during pregnancy	21 (0.1%)	0	0	–	–
Gestational diabetes	6 (0.04%)	1 (0.04%)	0	1.000	–
History of infection or drug application within the mother's third trimester of pregnancy	5 (0.03%)	0	0	–	–
Congenital diseases (including birth defects)	8 (0.05%)	2 (0.08%)	1 (0.4%)	0.647	0.156

^1^ *P*1-values are for the normal group compared with the group with RPD group.

^2^ *P*2-values are for the normal group compared with the group with moderate to severe RPD group.

The results of further multivariate logistic regression analysis of the statistically different factors showed that male (OR = 1.966, 95% CI: 1.791–2.158, *P* < 0.001), preterm (OR = 1.228, 95% CI: 1.102–1.367, *P* < 0.001) and twins (OR = 1.218, 95% CI: 1.098–1.351, *P* < 0.001) were risk factors for the occurrence of RPD in high-risk children, and macrosomia (OR = 0.698, 95% CI: 0.582–0.836, *P* < 0.001) was a protective factor for the occurrence of RPD in high-risk children (Table 4). Also, male (OR = 2.570, 95% CI: 1.932–3.420, *P* < 0.001) was a risk factor for the occurrence of moderate to severe RPD in high-risk children (Table 5).

**Table 4:** Multivariate logistic analysis of risk factors for RPD.

Factors	B value	Standard error	OR value	95% CI	*P*-value
Constants	−2.357	0.066	–	–	–
Male	0.676	0.048	1.966	1.791–2.158	<0.001
Premature	0.205	0.055	1.228	1.102–1.367	<0.001
Dystocia	−0.873	0.465	0.418	0.168–1.039	0.060
Twins	0.197	0.053	1.218	1.098–1.351	<0.001
macrosomia	−0.360	0.092	0.698	0.582–0.836	<0.001
Low birth weight	0.090	0.048	1.094	0.997–1.201	0.058

**Table 5:** Multivariate logistic analysis of risk factors for moderate to severe RPD.

Factors	B value	Standard error	OR value	95% CI	*P*-value
Constants	−4.533	0.187	–	–	–
Male	0.944	0.146	2.570	1.932–3.420	<0.001
Premature	−0.206	0.151	0.814	0.606–1.094	0.172
Dystocia	−0.551	1.016	0.576	0.079–4.220	0.587
Twins	0.287	0.149	1.333	0.995–1.786	0.054
macrosomia	0.013	0.212	1.013	0.669–1.535	0.950
Low birth weight	−0.356	0.140	0.701	0.532–0.922	0.011

## 5. Discussion

In this study, we summarized and analyzed 16,468 high-risk children who were screened with postnatal urological ultrasound through the three-level referral system from September 2010 to December 2019, the results showed a linear increasing trend in screening rate over a decade, especially after 2014, with a positive rate of RPD remaining at about 15% and screening volume remaining at about 1,500 cases per year. The referral and follow-up rates were significantly improved after 2015 after training and education of primary care physicians and parents. In addition, we performed univariate and multivariate logistic regression analyses on the occurrence of RPD in high-risk children, and the results showed that male, preterm and twins were risk factors for the occurrence of RPD in high-risk children, and male was also a risk factor for the occurrence of moderate to severe RPD in high-risk children.

CAKUT is the common birth defect and the leading cause of end-stage renal disease in children and adolescents (5, 12). However, CAKUT has an insidious onset and requires ultrasound screening or other tests to be detected. Early detection and early intervention can slow down disease progression. Therefore, we have created a three-level screening and referral system based on China's national conditions: community - district-level MCH – children's hospitals (9, 13). The analysis of the screening data over the past ten years from September 2010 to December 2019 showed that the three-level screening and referral system was stable and gradually smooth, especially after 2015, the screening rate, referral rate and follow-up rate improved significantly after training of primary care physicians and education of parents. Therefore, regular training and education for primary care physicians and parents are needed in the future. Japan, Germany, the Czech Republic, and Italy rely on their own national systems for early postnatal ultrasound screening. In Japan, early postnatal ultrasound screening is offered to 1-month-old children who come to the hospital for a health checkup (14, 15). In Germany, early postnatal ultrasound screening is offered to children 3 days after delivery in the hospital (16). Czech Republic provides ultrasound screening for children 72–144 h after delivery in our hospital (7). In Italy, early postnatal ultrasound screening is performed for children 2 months of age who come to the hospital for a health check-up (6). Our three-level screening referral system is based on China's national conditions, where the community can provide basic medical care and education, district-level MCH can perform ultrasound examinations, and Grade IIIA children's hospitals have specialized renal and urological doctors for diagnosis and treatment. Therefore, the three-level screening system for the urinary system, which has been operating smoothly for the past ten years, is in line with the Chinese national conditions.

In CAKUT, RPD from various causes is the most common and can be detected by ultrasound screening. Several studies have shown that most mild RPD returns to normal at follow-up, while moderate to severe RPD has a higher probability of diagnosing obstructive nephropathy (10, 11). Therefore, we further investigated the risk factors for the occurrence of RPD as well as moderate to severe RPD. In the present study, we explored the factors according to the *requirements for the management of high-risk children (low birth weight children) in Shanghai*, and found that male, preterm and twins were risk factors for the occurrence of RPD in high-risk children, and that macrosomia was a protective factor for the occurrence of RPD in high-risk children. Moreover, males were also risk factors for moderate to severe RPD in high-risk children. Previous studies have shown that preterm, twins, is closely associated with low birth weight, and that macrosomia is a protective factor for the occurrence of RPD in high-risk children. Therefore, it is presumed that the occurrence of RPD in high-risk children is closely related to birth weight and should be closely monitored in later screening. Furthermore, males are a risk factor for the occurrence of RPD and moderate to severe RPD in high-risk children, presumably due to longer male urethra anatomically. In addition, the results also indicated that low birth weight is a protective factor for the occurrence of moderate to severe RPD in high-risk children, which is inconsistent with previous studies, presumably because this study was a study based on a specific population and thus the conclusions may be influenced. Several studies have also reported low birth weight (17, 18), preterm birth (17, 19) is associated with the occurrence of CAKUT, Melo. et al. (19) found that preterm birth and low birth weight are independent risk factors for early death in patients with CAKUT, due to the lack of detailed weight data in this study, it is not yet possible to delineate the weight threshold, which requires more exploration in future work. In addition, factors such as the oligohydramnios (17, 19), hypothyroidism during pregnancy (19), maternal gestational diabetes (17, 18) and maternal overweight/obesity (18) have also been reported to be associated with the occurrence of CAKUT in children, which have not been included in this study and need improvement in future work.

Overall, this study demonstrated the gradual maturation and stability of the three-level referral system for urological ultrasound screening in Minhang District, Shanghai, through data analysis of the last decade, including screening volume, screening rate, referral rate and follow-up rate. Meanwhile, the risk factors for RPD and moderate to severe RPD were obtained by chi-square test and multivariate logistic regression analysis, which pointed out the key directions for future screening. However, health economics analysis was not performed in this study, the follow-up data were not complete, and the clinical factors included in the maternal and neonatal periods were not sufficiently comprehensive, which need to be improved in future work. Moreover, the present study only screened high-risk children born in the last decade by ultrasound, and the occurrence of renal pelvic dilatation in other children needs further observation. In the future, we hope that increasing postnatal ultrasound and eventually routine prenatal ultrasound should eventually detect the vast majority of children with renal pelvis dilatation that require surgery rather than waiting for clinical symptoms often at which time significant impairment of renal function has occurred.

## 6. Conclusions

The screening data of the recent ten years show that the three-level urological ultrasound screening referral system of community health centers-district maternal and child health hospitals-children's specialized hospitals has been operating steadily and gradually getting better. Univariate and multivariate logistic regression analyses showed that male, preterm and twins were risk factors for high-risk infants with renal pelvic dilatation, and male was also a risk factor for moderate to severe renal pelvic dilatation in high-risk infants.

## Data Availability

The original contributions presented in the study are included in the article, further inquiries can be directed to the corresponding author.
